# Diagnostic Quandary: *Salmonella* Agbeni Vertebral Osteomyelitis and Epidural Abscess

**DOI:** 10.1155/2018/1091932

**Published:** 2018-09-20

**Authors:** Ryan K. Dahlberg, Mary Elizabeth Lyvers, Thomas K. Dahlberg

**Affiliations:** ^1^University of Illinois College of Medicine, Rockford, USA; ^2^Rockford Pain Center, Rockford, USA

## Abstract

Salmonella vertebral discitis/osteomyelitis is a rare manifestation of Salmonella infection. Here, we report a case of a 54-year-old Caucasian male who presented with five weeks of progressively worsening bilateral low back, buttock, and lower extremity pain following an 8-foot fall onto concrete from a ladder. Initial workup following the fall included hip X-ray and MRI of the lumbar spine and revealed only mild lumbar facet arthropathy and moderate left neural foraminal stenosis at L3-L4 without any concomitant hip or spine fracture. The patient's pain continued to increase in severity over the next several weeks, and he was evaluated by multiple healthcare professionals with no discovered pathology. Approximately 5 weeks following the fall, repeat CT scan and MRI were conducted which then revealed extensive findings of discitis/osteomyelitis at L5–S1 as well as an epidural abscess resulting in severe narrowing of the central spinal canal. Patient underwent emergent decompression laminectomy and discectomy at L5–S1 with evacuation of the epidural abscess. Intraoperative tissue and wound cultures revealed Salmonella enterica serovar Agbeni. The patient recovered well and was discharged on an eight-week regimen of IV ceftriaxone. He has since recovered appropriately with no neurologic deficits. Important takeaways from this case include continuing to work up patients whose pain or condition is not consistent with radiographic findings and the importance of clinical intuition. This case also highlights the use of intraoperative cultures and sensitivities to correctly direct antibiotic management. Lastly, this report adds to the paucity of literature surrounding Salmonella Agbeni-related discitis and epidural abscesses and makes the suggestion that traumatic incidents such as a fall may instigate these infections.

## 1. Introduction

Nontyphoid Salmonella represents a subset of Salmonella infections that most commonly results in gastrointestinal illness, but has been shown to cause a spectrum of illnesses ranging from mild gastroenteritis to complications of septicemia. Salmonella has been recognized as an uncommon cause of vertebral osteomyelitis for over 100 years [1]. However, original reports were seemingly associated with typhoid Salmonella. It was not until 1957 that a study reported evidence of osteomyelitis as the result of nontyphoidal strains [2]. In 2017, an outbreak of Salmonella serovar Agbeni was reported in North America in which 73 individuals reported illness and 30 individuals were hospitalized [3]. In this report, we describe a case of a 54-year-old male with *Salmonella* ser. Agbeni vertebral osteomyelitis, discitis, and epidural abscess following a traumatic fall.

## 2. Case Report

**Figure 1:**
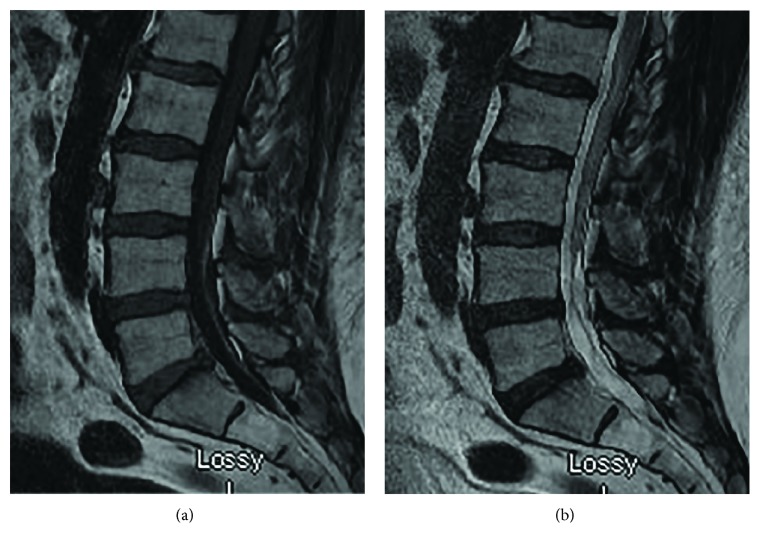
T1- and T2-weighted sagittal images of the lumbar spine revealing no acute processes. This study is from the initial workup in the emergency dept.

A 54-year-old Caucasian male with past medical history positive only for diverticulitis with a resultant sigmoidectomy presented to a pain management specialist with progressively worsening bilateral low back, buttock, and radicular leg pain, weakness, and numbness for five weeks after falling from an 8 ft. ladder onto concrete. At the time of the fall, no notable injury was sustained and the patient reported only minimal low back pain without radiculopathy. Over the next several days, the patient began to experience numbness and weakness of the bilateral lower extremities. Evaluation in the ER, which included a hip X-ray and MRI of the lumbar spine (seen in Figure 1), revealed only mild lumbar facet arthropathy and moderate left neural foraminal stenosis at L3-L4 without any concomitant hip or spine fracture.

**Figure 2:**
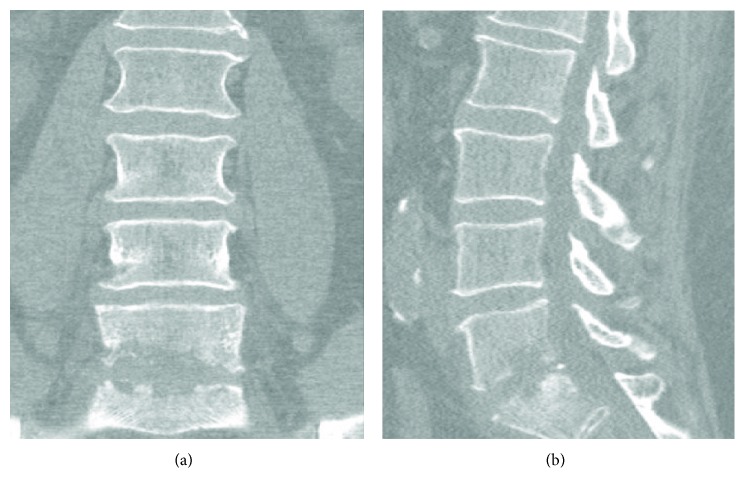
CT imaging of lumbar spine approximately 5 weeks after initial fall. Evidence of significant discitis and end plate destruction at L5/S1 level.

**Figure 3:**
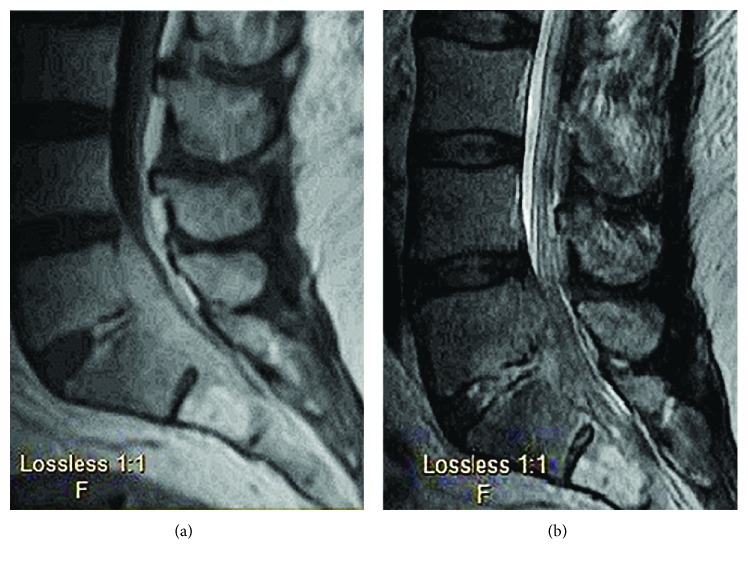
Post-T1- and T2-weighted MRI of lumbar spine upon hospital admission. Confirmed suspected discitis, osteomyelitis with end plate destruction, and epidural abscess seen on CT.

Following discharge, the patient reported worsening low back pain and bilateral lower extremity weakness in both L5 and S1 distributions. Several weeks after the initial fall, the patient was referred to pain management where he was evaluated and eventually underwent a radionuclide bone scan. This revealed mildly increased uptake at L5/S1 but was suspected to be a result of facet osteoarthritis. The patient denied any fever at this time, constitutional symptoms, or recent illness. Review of symptoms and physical exam were unremarkable aside from mild paraspinal tenderness and lumbosacral radiculopathy. The rest of the patient's neurologic exam was normal. The patient was instructed to follow up in one week's time. At the return visit, he reported continued worsening of symptoms with no relief. At this point in time, the patient mentioned that he had been experiencing shaking chills at night. Other review of symptoms and physical exam remained unchanged, and the patient was noted to be afebrile. A CT scan (Figure 2) and subsequent MRI of the lumbar spine (Figure 3) were conducted which revealed extensive findings of discitis/osteomyelitis at L5–S1 as well as an epidural abscess resulting in severe narrowing of the central spinal canal. An emergent decompression laminectomy and discectomy was performed at L5–S1 with evacuation of the epidural abscess. Intraoperative tissue and wound cultures revealed Salmonella enterica serovar Agbeni. The patient recovered well and was discharged on eight weeks of IV ceftriaxone as dictated by culture sensitivities. Patient has recovered well with no neurologic deficits.

## 3. Discussion

Salmonella has long been recognized as a causative organism of vertebral osteomyelitis [1, 2]. However, this case represents the first known report of such a manifestation by the Agbeni serovar. Common presenting symptoms of this infection include fever and back pain [4]. This patient was not noted to be febrile until what was thought to be late in the disease course. Not only is this a unique manifestation of the bacteria but also correlates well with the 2017 Salmonella outbreak reported by the CDC [3]. This further underlines the importance of remaining continuously well-informed with respect to current epidemiologic trends, regardless of practice scope.

A review by Santos and Sapico reveal that vertebral osteomyelitis most commonly occurs at the lumbar level (50% of cases) and that a majority (54%) of patients exhibit some predisposing factor for the infection including atherosclerosis, sickle cell disease, diabetes, collagen diseases, liver cirrhosis, achlorhydria, or turtle contact (in the case of Agbeni specifically) [3–5]. The review goes on to state that trauma was not reliably linked to Salmonella infection risk. While the patient outlined in this report did have lumbar involvement, he exhibited no other risk factors for the Salmonella infection. He did, however, fall from the ladder onto his back, resulting in closed trauma without radiographic evidence of injury. This, coupled with his history of diverticulosis, a disease implicated in at least two other reports of non-Salmonella vertebral osteomyelitis, makes a case for trauma being involved in the etiology of this patient's infection [6]. A report in 2016 by Khoo et al. additionally described a 56-year-old male with no risk factors for Salmonella osteomyelitis who fell onto his back and subsequently developed Salmonella typhi vertebral osteomyelitis [7]. This should at least suggest the possibility of trauma as implicated in the seeding of these typically gastrointestinal bacteria either directly to the spine or hematogenously. This case we report is interesting though, in that the history of the fall may have actually delayed the diagnosis and made it that much more difficult to discern the etiology of the pain. The initial workup following the fall came back negative for any acute processes. Therefore, the patient's pain was continuously attributed to a myofascial or skeletal sequela of his fall. This serves as an important lesson in clinical intuition. When a patient's pain or presenting symptoms are not consistent with radiographic or lab findings, it is important to continue to work the patient up and maintain a broad differential.

Salmonella osteomyelitis and abscess can primarily be addressed with a combination of debridement strategies and antibiotics. Patients with superficial abscesses may only require aspiration whereas cases with extensive bone involvement and epidural abscesses may require significant debridement and drainage [4]. In the past, antibiotics of choice for treatment included trimethoprim-sulfamethoxazole, amikacin, and cefotaxime. More recently, reports have shown success with fluoroquinolones, with ampicillin and 3rd generation cephalosporins as alternatives due to increased recognition of antibiotic resistance. The review by Santos and Sapico suggested total duration of antibiotics for longer than 8 weeks regardless of antibiotic [4]. In addition to the laminectomy and abscess drainage, the above patient's antibiotic regimen of IV ceftriaxone was directed by intraoperative culture sensitivities and was continued for a total of 8 weeks. This highlights the importance of cultures and sensitivities for not only definitively diagnosing a Salmonella infection but also directing appropriate antibiotic management and stewardship.
